# Patient doses in image-guided radiotherapy: status in Europe for cone-beam CT imaging in the pelvic region

**DOI:** 10.2478/raon-2026-0012

**Published:** 2026-03-24

**Authors:** Toni Kansanoja, Serhii Brovchuk, Milana Vezirovic, Borislava Petrovic, Antonio Giuseppe Amico, Sonia Sapignoli, Marta Paiusco, Paolo Ferrari, Ana Cravo Sá, Anabela G Dias, Pedro Teles, Teemu Siiskonen

**Affiliations:** Department of Physics, University of Helsinki, Helsingin Yliopisto, Finland; Helsinki Institute of Physics, Helsingin Yliopisto, Finland; LISOD Israeli Oncology Hospital, Pliuty, Ukraine; State Institute “O.O. Shalimov Scientific Center of Surgery and Transplantation”, Kyiv, Ukraine; Department of Physics, Radiotherapy Department, University of Novi Sad, Novi Sad, Serbia; Oncology Institute of Vojvodina, Sremska Kamenica, Serbia; Medical Physics Department, Veneto Institute of Oncology IOV-IRCCS, Via Gattamelata 64, 35128 Padova, Italy; ENEA, Istituto di Radioprotezione, Radiation Protection Institute, Bologna, Italy; Centro de Ciências e TecnologiasNucleares (IST), Bobadela LRS, Portugal; TBIO|RISE-Health, Escola Superior de Saúde - Politécnico Porto, Porto, Portugal; Institute Português de Oncologia do Porto Francisco Gentil (IPOPFG) - Medical Physics Service, R. Dr. António Bernardino de Almeida, Porto, Portugal; Medical Physics, Radiobiology and Radiation Protection Group, IPO Porto Research Centre (CI-IPO), Portuguese Oncology Institute of Porto (IPO-Porto), Porto Comprehensive Cancer Center (Porto.CCC) & Rise@CI-IPOP (Health Research Network), Porto, Portugal; Department of Physics and Astronomy of the Faculty of Sciences of the University of Porto, Rua do Campo Alegre, Porto, Portugal; Radiation and Nuclear Safety Authority (STUK), Vantaa, Finland

**Keywords:** CBCT, image-guided radiotherapy, optimization, organ dose, diagnostic reference level (DRL)

## Abstract

**Background:**

Organ absorbed doses in cone-beam CT (CBCT) imaging are often neglected in image-guided radiation therapy (IGRT). However, frequent imaging for patient positioning can result in significant and unrecorded additional radiation exposure. This study aims to evaluate organ doses from kV-CBCT and assess if they are optimized and how, in prostate and pelvic patient positioning protocols across Europe. Status of quality assurance in IGRT CBCT imaging is assessed in general.

**Materials and methods:**

Data collected from a survey distributed across Europe on IGRT practices were compiled and analysed. A representative set of imaging protocols were simulated using Monte Carlo based ImpactMC software to assess mean absorbed doses in various organs in the International Commission on Radiological Protection (ICRP) standard phantom. Absorbed doses to red bone marrow were estimated with a three-parameter mass-energy absorption coefficient method. The simulations were validated against measurements with MOSFET detectors and radiochromic film.

**Results:**

Simulated prostate absorbed doses ranged from 12 mGy to 34 mGy per imaged fraction for pelvic protocols, and 4 mGy to 26 mGy for prostate protocols. The selected length of the imaging region influenced doses to the femur and sacral red bone marrow. Overall, 74% of treatments involved positioning imaging at every fraction, indicating substantial cumulative doses from kV-CBCT imaging. Quality assurance was performed by 90% of responders, but good practice guides and national protocols do not exist.

**Conclusions:**

The results of this study suggest that clear guidelines and standardized protocols for CBCT imaging in IGRT are lacking. There is significant potential to optimize the patient doses resulting from imaging. Given that most clinics already perform regular quality assurance for imaging equipment, including dosimetry and positioning accuracy verification, establishing diagnostic reference levels for CBCT imaging in IGRT could help promote further dose optimization.

## Introduction

Anatomical variations, such as organ motion or deformation, will occur in the patient during the radiation therapy process, which may lead to discrepancies between the calculated and delivered dose distributions, resulting in either larger (than planned) doses to healthy tissues or under dosing of the planning target volume. Throughout treatment course, this may compromise the effectiveness of treatment and increase the risk of unwanted side effects or recurrences. It is known that 5% deviation in the prescribed treatment dose may induce significant alterations in tumour control probability and increase the risk to adjacent organs at risk.^1^

The discrepancy between planned and delivered doses due to anatomical variations is significantly mitigated using image-guided radiotherapy (IGRT), which ensures accurate positioning and consequently radiation beam delivery through various imaging techniques performed immediately before the treatment. For this purpose, one of the most common techniques used is cone beam computed tomography (CBCT), but also projection radiography using kV or MV photons, magnetic resonance imaging (MRI), ultrasound, surface imaging, and positron emission tomography are used.^2^ The CBCT technique has almost fully replaced traditional projection imaging and MV beam imaging and became widely accessible technique and is the predominant modality employed globally.^3,4^

Frequent and non-optimized imaging exposes patients to additional radiation risk. Optimization involves careful selection of parameters such as the length of the imaged region, X-ray tube current and voltage, field of view, number of projections, and the use of X-ray beam filtration, while still ensuring acquisition of the necessary patient-positioning information.^5^ Typically, the imaged region extends several centimetres, or more, beyond the treated area, thereby exposing volumes of healthy tissue to substantial doses if imaging is performed at every treatment fraction (up to 40 fractions, although nowadays often fewer).^6-9^ Additionally, the dose from imaging contributes to the target volume dose but also dose to organs-at-risk.

Despite their routine use, radiation doses associated with patient positioning imaging have traditionally received little attention. However, awareness of the need to optimize the dose from imaging in IGRT has increased in recent years.^10-13^ Recognizing the importance of the issue, the International Commission on Radiological Protection (ICRP) has established a task group to advance radiological protection aspects of imaging in radiotherapy (ICRP Task Group 116, https://www.icrp.org/icrp_group.asp?id=182). This group presented a methodology to apply correction factors on Varian TrueBeam linacs without requiring 100 mm chambers or cylindrical phantoms, enabling centres worldwide to perform meaningful imaging dose measurements and optimisation.^4^

The European Radiation Dosimetry Group (EURADOS, https://eurados.sckcen.be) promotes research, development, and European cooperation in ionizing radiation dosimetry. EURADOS contributes to the harmonization of dosimetric procedures within the EU and ensures their alignment with international standards. To assess the status of patient positioning imaging in Europe and to promote the optimization of patient exposures, EURADOS launched a project to investigate imaging practices across Europe and to estimate corresponding patient organ doses in selected treatments. For this purpose, a questionnaire was developed and distributed via EURADOS contact persons to radiotherapy departments. The data presented in this study pertain to treatments in the pelvic region, with a particular focus on imaging for prostate treatments.

Diagnostic reference levels (DRLs) are used in diagnostic imaging to support the optimization process. DRLs serve as guidelines for evaluating patient radiation doses during specific procedures, helping to identify whether a dose is unusually high or low. This facilitates the optimization of radiation exposure while ensuring the necessary image quality and diagnostic information. Although DRLs are not traditionally applied to radiation therapy, the ICRP recommends their use for patient setup verification imaging in radiotherapy.^14^ While this study does not aim to establish regional DRLs for patient positioning imaging, its results provide an indication of typical exposure levels and thus serve as reference and benchmark for future studies focused on establishing national or regional DRLs.

## Materials and methods

### Questionnaire on imaging practices and quality assurance

The questionnaire was developed to analyse current imaging practices in radiotherapy clinics across Europe. It was distributed via email to radiotherapy centres, national societies, and through EURADOS Working Group on Radiotherapy Dosimetry. Direct contacts were established with 16 European countries. The questionnaire was distributed in September 2023, and the final responses were collected in April 2024.

The questionnaire was structured in two parts. The first part focused on general institutional data and practice, including the number and type of radiotherapy machines and their age, quality assurance protocols for imaging devices, recording of doses from imaging, and the frequency, and type of imaging performed for different anatomical sites and patient age groups (adults vs. paediatric patients).

The second part of the questionnaire focused on gathering detailed information about the specific radiotherapy machines identified in the first section. Participants were asked to select up to two machines that best represent clinical practice across different treatment sites, with particular emphasis on the pelvic region. This section of the questionnaire aimed to obtain detailed information on the use of projection imaging performed with the treatment beam and with a dedicated X-ray device (planar MV and kV imaging), as well as on volumetric imaging (MV and kV CBCT imaging) carried out during radiotherapy treatments.

Specific questions addressed the frequency and the type of imaging for each treatment site, adjustment of field size, imaging protocol and dose recording practices, X-ray tube settings (current and voltage U), pulse length, number of projections, weighted CT dose index (CTDI_w_)^15^, dose normalization (CTDI_w_/100 mAs), gantry speed, rotation angle, field of view (FOV), length of the imaged region, geometric distances (e.g., focus-to-isocentre and focus-to-detector), acquisition time, and the filter type (full fan or half fan).

To ensure anonymity, the results were coded: the letter indicates the country, while the sequential number identifies the specific radiotherapy clinic or hospital within that country.

### Monte Carlo calculation of organ doses

#### General method

Computational results presented in this study were computed using ImpactMC (version 1.20^16^). It uses DICOM files as input to represent patient anatomy. Output is a three-dimensional dose distribution in the DICOM file geometry.

Monte Carlo simulations used technical parameters of imaging protocols obtained from the questionnaire. These protocols were grouped based on the imaged site (e.g. pelvis or prostate), X-ray tube voltage and current, and X-ray beam collimation width. As most hospitals reported similar imaging protocols, the data were consolidated to retain only a few representative cases. Only pelvis and prostate imaging protocols were simulated.

#### Simulated protocols

Key parameters for simulated pelvic and prostate protocols are presented in Table 1 and Table 2, respectively.

**TABLE 1.** Parameters for pelvic area kV cone-beam CT (CBCT) simulations. All protocols employ half fan filtering unless otherwise stated. The last protocol shows the parameters used in validation measurements

Hospital	Machine manufacturer/model	U (kV)	Q (mAs)	CTDIw (mGy)	Projections	X-ray source start-stop angle (degrees)	Imaged region length (cm)	FOV (cm)	Notes
**C**	Elekta Agility	120	1056	22.0	660	-180 to 180	41	27	Vendor provided
**G1**	Elekta Agility	120	528	11.0	330	90 to -89	28	42	Vendor provided
**A3**	Varian ClinaciX	125	659	17.8	650	182 to - 178	11	30	Vendor provided
**D1**	Varian TrueBeam	125	1080	16.0	900	90 to 270	15	45	Vendor provided
**F1**	Varian Trilogy	125	697	17.8	670	180 to -180	16	45	Vendor provided
**F6**	Varian Halcyon	125	592	11.8	529	-180 to 180	22	21	Vendor provided
**H1 60mA**	Varian TrueBeam	125	900	13.3	900	180 to -180	21	45	Full fan filter. Optimized on site
**H1 80mA**	Varian Halcyon	125	1074	21.5	895	181 to -181	25	45	Vendor provided
**O2**	Varian TrueBeam	125	684	11.5	900	Full rotation	16	45	Start-stop angles assumed
**-**	Varian TrueBeam	125	1080	-	900	180 to 180	17	-	Protocol used in MOSFET and LD V1 measurements

CTDI_w =_ weighted CT dose index; FOV = field of view

**TABLE 2.** Parameters for prostate imaging kV cone-beam (CBCT) protocols. All protocols employ half fan filtering

	Machine manufacturer and model	U (kV)	Q (mAs)	CTDIw (mGy)	Projections	X-ray source startstop angle (degrees)	Imaged region length (cm)	FOV (cm)
**G1**	Elekta Agility	120	528	11.0	330	90 to -89	28	42
**M1**	Elekta VersaHD	120	845	12.2	660	-180 to 180	21	28
**M4 narrow**	Elekta Synergy	120	422	5.7	330	-180 to 180	14	28
**M4 wide**	Elekta Synergy	120	422	7.8	330	-180 to 180	28	28
**F2**	Varian TrueBeam	125	1080	16.0	900	180 to -179	18	49
**J**	Varian TrueBeam	125	252	3.70	900	-179 to 181	16	45

CTDI_w =_ weighted CT dose index; FOV = field of view

The X-ray beam shaped filter (bowtie filter), and the X-ray spectrum were given as an input for ImpactMC. X-ray tube energy spectra were computed using the SpekPy code.^17^

Three bowtie filter profiles were used: one for Elekta half-fan setup, and two for Varian (halffan and full-fan setups). Differences between individual machine models may introduce small inaccuracies in the results. However, previous work showed that bowtie filters used in Varian TrueBeam models were similar.^8^ Therefore, it was assumed here that the differences between models from the same manufacturer would not introduce significant uncertainty.

Air kerma free-in-air at isocentre (K_a_) required by ImpactMC was simulated from the CTDI_w_ values with 32 cm diameter CTDI phantom using full fan geometry with 16° fan angle and 10 cm total beam collimation.

Full-fan beam geometry was specified in ImpactMC using the fan angle parameter. This angle was estimated based on the source-to-isocentre and source-to-detector distances, the field of view, and the imaging length. It was further assumed that the beam would fully cover the detector, and that at the isocentre, the beam would deviate by at most half of the field of view from the isocentre.

#### Dose calculation

In the simulations, ICRP’s Computational Reference Phantom (CRP) was used as the anatomical model of the patient.^18^ The CRP was modified by removing arms to replicate a realistic imaging setup where arms are kept outside the X-ray field. For ImpactMC, the material composition of the CRP was expressed in Hounsfield units rather than the elemental compositions of the original CRP. At least 10^10^ source photons were simulated to ensure sufficient statistical accuracy in organ dose calculations.

The absorbed dose for each organ in CRP was calculated as the mean absorbed dose across all voxels assigned to that organ. The uncertainty associated with each organ dose was estimated using the bootstrapping method. The analysis focused on organs near the X-ray beam centre and on those with the highest tissue weighting factors.^19^

The absorbed doses to red bone marrow (RBM) were estimated using the ratios of mass-energy absorption coefficient ratios and dose enhancement factors in the RBM and the surrounding spongious bone, the so-called three-parameter MEAC method.^20,21^ Mass-energy absorption coefficient ratios between spongiosa volume of pelvis, femora and sacrum and RBM, and dose enhancement factors for active bone marrow were obtained and interpolated from Johnson *et al*.^20^

The initial X-ray tube spectrum was used as an estimate of the photon spectral fluence at the RBM site. Thus, the RBM dose was obtained by multiplying the dose to the bone in CRP by photon spectrum weighted averages of mass-energy absorption coefficient ratios and dose enhancement factors.

### Dose measurements in anthropomorphic phantom

Dose measurements using radiochromic films and MOSFET detectors were performed to validate the Monte Carlo (MC) simulation. The measurements employed MOSFET (mobileMOSFET, Best Medical, Canada) and LD-V1-1012 radiochromic film (Ashland Advanced Materials, Bridgewater NJ, USA) within a pelvic anthropomorphic phantom (ATOM CIRS Adult Male Model 701).

The detectors were calibrated in air with a stationary CBCT X-ray tube^13^, using a RaySafe (X2 R/F 305675) detector as a reference, calibrated by the Swedish Board for Accreditation and Conformity Assessment. As suggested by Brady *et al*.^22^, a correction factor was established to account for differences in scatter, geometry, and beam modulation between calibration and measurement conditions. For this purpose, each MOSFET and a pencil ionization chamber (RaySafe X2 CT 293677) were positioned at the centre of a 32 cm CTDI phantom.

**FIGURE 1.**
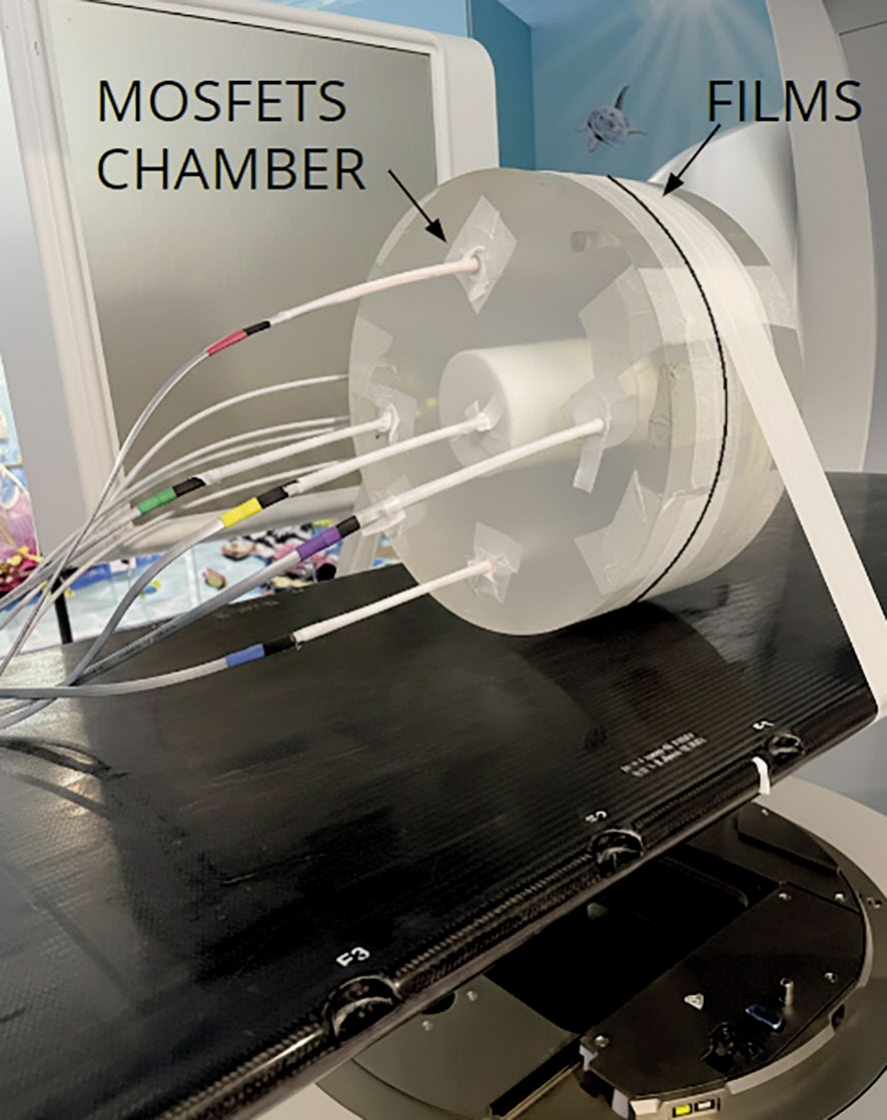
Positions of LD-V1 films and MOSFETs in the CT dose index (CTDI) phantom.

To ensure consistency of measurements, a dose comparison between MOSFETs, films (sandwiched between two CTDI phantom slabs), and the pencil ionization chamber, with each detector positioned sequentially in the CTDI phantom (3, 6, 9, 12 o’clock, and the phantom centre) was done as in Figure 1.

**FIGURE 2.**
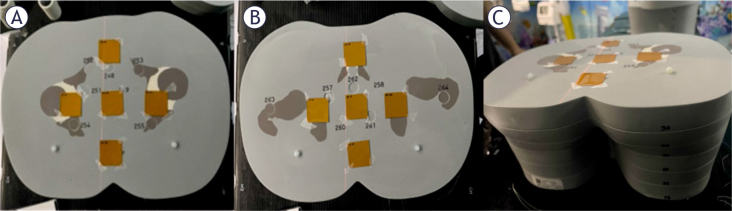
Positions of LD-V1 films in the anthropomorphic phantom. Panel **(A)** shows film positions between slabs 33 and 34, and panel **(B)** between slabs 34 and 35. Panel **(C)** shows a side view of the phantom with films on top of slab 34 (corresponding to panel **(A)**).

The LD-V1 film’s response to the irradiation direction was evaluated under two configurations: (i) with the beam axis aligned parallel to the film surface, and (ii) with the beam axis-oriented perpendicular to the film surface. Validation measurements were performed by averaging doses from four CBCT scans of the anthropomorphic phantom, using the scanning protocol of Table 1. Ten LD-V1 film pieces (3 cm × 3 cm) were placed between phantom slabs, as illustrated in Figure 2. Five films were positioned between slabs 33 (Figure 2A) and 34, and another five films between slabs 34 (Figure 2B) and 35. Slab 34 was located above the isocentre plane, while films on slab 35 were aligned with the isocentric plane. In both positions, one film was placed in the centre, and four films at 3, 6, 9, and 12 o’clock positions with about 3 cm spacing between them.

Figure 2C presents an image of the phantom slab numbers, where the films are attached to the surface of slab 34. The dose was obtained as a mean dose in the central 2 cm x 2 cm region of the film.

**FIGURE 3.**
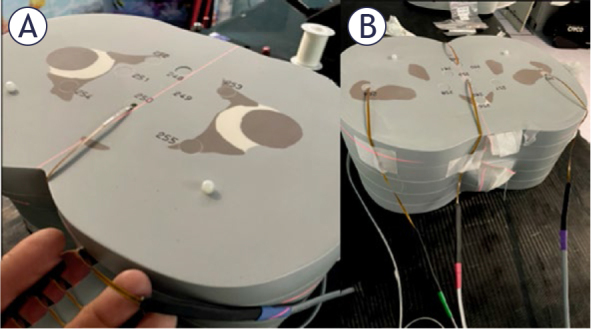
Positions of MOSFET detectors in the anthropomorphic phantom. Left panel shows the detectors in slab number 34 **(A)** and the right panel in slab number 35 **(B)**.

Similarly, in separate scans, five MOSFET detectors were placed inside slabs 34 and 35 (Figure 3) at centre (prostate), posterior, anterior and lateral positions.

The Varian TrueBeam setup used in the phantom measurements was modelled in ImpactMC. These CBCT simulations were done without detectors to avoid any perturbations they might introduce to the dose distribution. Simulated mean absorbed doses were obtained in regions of interest (ROIs), corresponding the locations of the film or MOSFET detectors. The statistical uncertainty of the simulated dose was estimated by analysing standard error of the mean in ROIs in adjacent, homogenous regions of the dose distribution.

## Results

### The questionnaire

**FIGURE 4.**
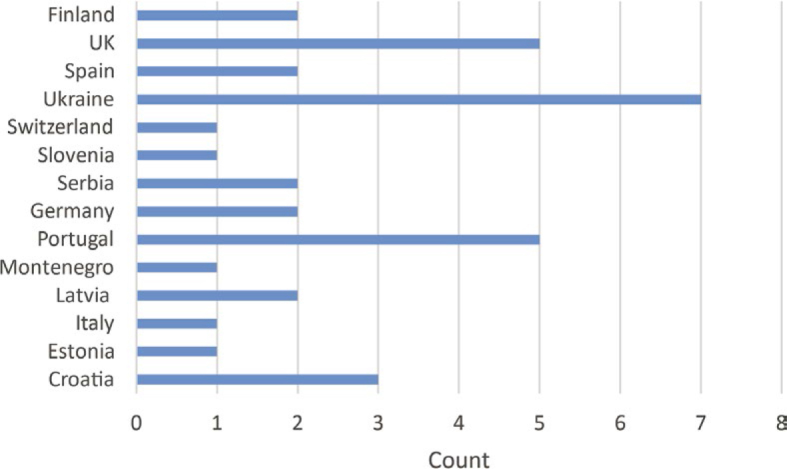
Number of answers to the questionnaire among countries.

In total, 35 hospitals (country distribution shown in Figure 4) participated in the survey, submitting data about 68 accelerators and 243 treatment sites. A single accelerator can be used to treat different anatomical sites (e.g., pelvis and head). Across all treatment sites, 115 kV-CBCT protocols were collected, of which 44 protocols considered prostate, pelvis or rectum.

**FIGURE 5.**
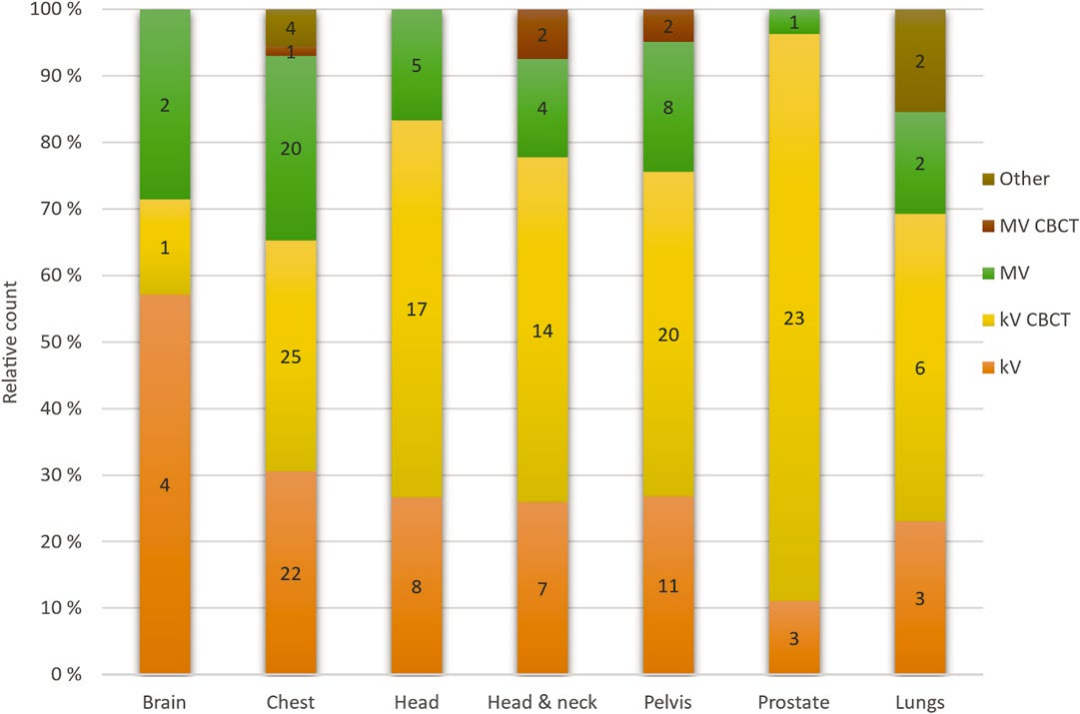
Number and relative amount (%) of different imaging modalities site by site. CBCT = cone-beam CT

Patient positioning imaging was used across all clinical sites. Treatment sites with more than four answers are shown in Figure 5. Sites with less than four answers were spine (3 answers), stomach (2 answers) and mediastinum (2 answers). In two cases, no treatment site was specified. kV-CBCT and planar kV imaging were the most widely used modalities. Other patient positioning techniques than kV/MV CBCT and planar imaging were surface-guided positioning, Calypso (Varian Medical Systems, Inc), and magnetic resonance imaging.

The majority (74%) of the reported treatment protocols involved imaging at every treatment fraction. The rest used imaging during the first one to three fractions (21%) or the first four to seven fractions (5%), followed by weekly imaging. Some clinics return to daily imaging if inconsistencies are observed. Out of nine clinics that reported treatment of paediatric patients, three used dedicated paediatric imaging protocols. Typical modification to the protocol when paediatric patients were treated was the reduction of the X-ray tube voltage and *Q*.

Patient dose from imaging was recorded in 11 hospitals (31%). The dose was obtained either from the treatment planning system or estimated using CTDI or CTDI_w_ values in the patient treatment records as a surrogate for the patient dose. Two hospitals reported that doses from imaging were explicitly included in the total treatment dose calculation.

The quality assurance of imaging devices was conducted by all but three clinics from 31 answers (excluding four blank answers). Quality assurance tests included isocentre positioning accuracy, image quality tests (tests for contrast, noise, and uniformity in the images; usually carried out monthly), and most notably, dose measurements.

Verification of the absorbed dose was typically done in the head or body CTDI phantom (13 hospitals). Measured dose quantities were CTDI_w_ or CTDI, in most cases using a 10 cm pencil ionization chamber. Other dose measurement methods were in-air kerma measurement with a Farmertype chamber, measurements in a slab phantom with a Farmer-type chamber, and measurements in an anthropomorphic phantom with an ionization chamber. Imaging dose verification measurements were typically done once per year. Image quality tests were mostly done with the Catphan phantom (Phantom laboratory, Salem NY, USA).

The survey collected information on national protocols or good practices in patient positioning imaging. Of the 32 respondents, 30 answered this question: only two clinics reported having national protocols in place. Two additional clinics from the same country indicated that they follow national protocols for quality assurance testing. Additionally, one hospital reported following regional protocols, and another reported following international protocols. The remaining 24 answers indicated that no guidelines are followed.

Less than half of protocols were optimized at hospitals: when imaging the pelvic region, 43% of the hospitals optimized their protocols from vendor-provided protocols (prostate imaging was optimized more often than generic pelvic protocol). In the thorax area, 41% of the protocols were optimized at hospitals. Only 17% of protocols in the head and neck area were optimized.

In the kV-CBCT protocols for pelvis and prostate imaging, X-ray tube voltages ranged from 120 kV to 125 kV, while tube current varied from 14 mA to 80 mA. The typical number of projections was 330, 660 or 900. Bowtie filters were employed for imaging of the trunk to reduce dose to superficial organs. The length of the imaging region ranged from 11 cm to 41 cm. The FOV ranged from 21 cm to 49 cm (Tables 1 and 2).

Tables 3 and 4 show the third quartiles of medians of CTDI_w_ values from the questionnaire (CTDI_w_ is recommended as a CBCT DRL quantity) for pelvic and prostate protocols, per each country. These third quartiles represent national DRL values.^14^ The indicative regional DRL is the median of national values, 17.7 mGy for pelvic and 13.5 mGy for prostate imaging protocols.

**TABLE 3.** Third quartiles of median reported weighted CT dose index (CTDI_w_ values by country for pelvic imaging protocols

Country	A	B	C	D	E	F	G	H	K	M	Median*
Third quartile CTDI_w_ (mGy)	17.8	16.9	22.0	16.0	37.6	15.6	11.0	17.7	21.7	22.0	**17.7**

* The median column shows the median of the third quartiles

**TABLE 4.** Third quartiles of median reported CT dose index (CTDI_w_ values by country for prostate imaging protocols

Country	C	D	F	G	H	J	L	M	O	Median*
Third quartile CTDI_w_ (mGy)	32.2	16.0	19.7	11.0	13.5	3.7	16.0	9.6	11.3	**13.5**

* The median column shows the median of the third quartiles

### Organ doses

Organ mean absorbed doses for sigmoid colon, rectum, prostate, stomach, bladder wall, and RBM of femora, pelvis, and sacrum are shown in Tables 5 and 6 for pelvic and prostate protocols, respectively.

**TABLE 5.** Mean absorbed doses (mGy) for the pelvic protocols, per hospital and imaged fraction. Relative standard uncertainty is 16% for red bone marrow (RBM), 13% for other organs

Pelvic protocols	Organ mean absorbed dose per imaged fraction (mGy)							
Hospital	Sigmoid colon	Rectum	Prostate	Stomach	Bladder	Femora RBM	Pelvis RBM	Sacrum RBM
**C**	39.8	38.1	34.1	1.3	39.2	31.9	35.9	36.2
**G1**	14.4	16.5	14.5	0.1	16.3	10.5	12.9	14.6
**A3**	13.5	20.8	15.1	0.0	20.1	7.7	10.0	5.7
**D1**	22.6	32.8	27.4	0.0	30.9	13.2	17.5	14.6
**F1**	10.1	14.4	12.3	0.0	13.7	5.9	7.9	7.2
**F6**	11.8	13.3	11.6	0.1	13.1	7.1	9.6	10.4
**H1 (60 mA)**	24.6	29.8	25.9	0.1	28.2	13.7	19.0	23.0
**H1 (80 mA)**	15.7	19.4	18.5	0.0	18.7	10.0	13.3	16.0
**O2**	16.9	23.9	20.2	0.0	22.5	9.9	13.2	12.0

**TABLE 6.** Mean absorbed doses (mGy) for the prostate protocols, per hospital and imaged fraction. Relative standard uncertainty is 16% for red bone marrow (RBM), 13% for other organs

Pelvic protocols	Organ mean absorbed dose per imaged fraction (mGy)							
Hospital	Sigmoid colon	Rectum	Prostate	Stomach	Bladder	Femora RBM	Pelvis RBM	Sacrum RBM
**G1**	13.2	15.4	15.1	0.0	16.9	11.9	11.6	10.1
**M1**	14.2	18.8	17.3	0.0	19.0	15.2	12.6	6.0
**M4 Narrow**	1.5	3.9	3.9	0.0	3.7	3.0	1.7	0.6
**M4 Wide**	5.9	6.5	5.8	0.0	6.6	5.4	4.4	4.3
**F2**	13.1	22.6	26.1	0.0	25.9	14.7	12.5	4.8
**J**	2.3	4.4	4.8	0.0	4.7	2.6	2.2	0.8

Organ doses show significant variation between protocols in the same target area. Tables 5 and 6 show that doses to the prostate fell between 4 and 34 mGy per imaged fraction, lowest values obtained with optimized protocols of hospitals M4 and J.

### Dose measurement data and simulation results comparison

Measurements with MOSFET, pencil ionization chamber and radiochromic films agreed with each other. The differences in measured doses between MOSFET and pencil ionization chamber were typically 3% (range from 1.8% to 10%). The differences between films and pencil ionization chambers in CTDI phantom were typically 1% (range from 0.0% to 2.7%).

**FIGURE 6.**
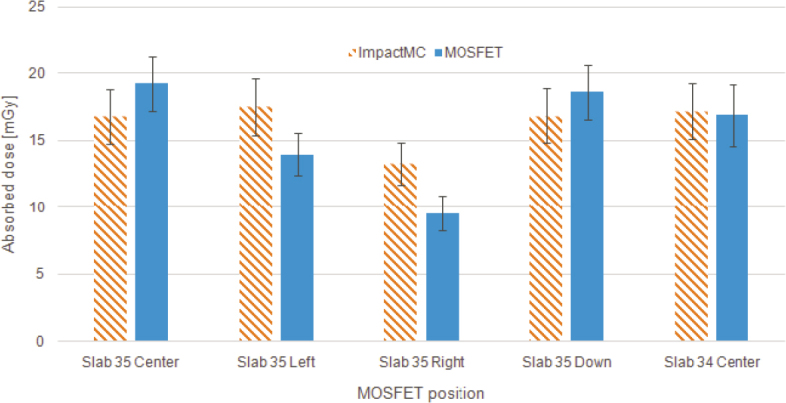
Comparison of MOSFET measurements in anthropomorphic phantom with simulated doses.

MOSFET measurements in anthropomorphic phantom agree within uncertainties with the simulated results in all cases except for right position (Figure 6). The largest discrepancies between MOSFET and simulated doses were in the right and left MOSFET positions at slab number 35 where the simulated mean doses in ROI were 39% and 25% higher than the measured doses. The doses at the centre of slabs number 35 and 34 and the down position agreed within 13%. In all positions except right the doses were compatible within the uncertainties.

**FIGURE 7.**
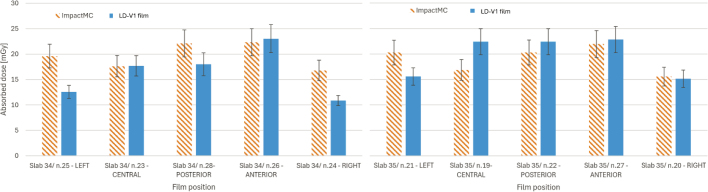
Comparison of LD-V1 film measurements in anthropomorphic phantom with simulated doses. Data for slab number 34 is shown in the left panel, for slab number 35 in the right panel.

The results of the film simulation show slightly larger differences with the measurements than with MOSFETs. Figure 7 shows differences between simulation and measurement in both slabs.

### ImpactMC model uncertainties

The relative statistical uncertainties in simulated organ doses were between 1-4% for organs in the primary beam. Major uncertainties in the simulations came from the conversion of CTDI_w_ to air kerma free-in-air, modelling of the beam geometry, and in the case of calculating doses from the measurement setup, the placement and size of ROIs (uncertainty in dose measurements was estimated to be 15% inside the phantoms). These uncertainties were assumed to be independent and were added in quadrature.

The conversion to air kerma free-in-air was simulated with ImpactMC. The resulting conversion factor has uncertainty from the manual positioning of the isocentre in the CTDI phantom, statistical uncertainty, and the used X-ray spectrum and bowtie filter. The exact shapes of the bowtie filters of individual machines of this study were unknown which resulted in additional uncertainty in the calculated conversion factors. This uncertainty was estimated to be less than 5%, as previous study showed that bowtie filters of Varian models were similar.^8^

The largest source of uncertainty in simulations stemmed from the beam geometry. Extending collimation from the inner side by 10 mm at the isocentre increased the dose by 10-20%. Widening the collimation at the isocentre from the outer side did not affect doses to other important organs other than the bladder. The bladder was the most prone to changes in collimation, since it is a small organ at the edge of the primary X-ray beam for most protocols, therefore the change in beam width affects its dose significantly. Here it is assumed that this contributes 10% to the uncertainty.

Change in the collimation in vertical direction increased the imaging region length, which in turn increased doses to organs at the edge of the beam. The beam geometry was defined within 5% accuracy which translates to a maximum of 8 mm changes in the collimation. Combining all uncertainty sources gives the simulations a total standard uncertainty of 13%. For RBM dosimetry there is an additional 10% uncertainty related to weighted mass-energy absorption coefficients and the fluence spectrum inside the phantom, yielding a total standard uncertainty of 16%. These uncertainties are associated with doses in CRP, and uncertainties in real patient anatomies may be larger.

## Discussion

The results show that there are significant variations in mean organ absorbed doses in the imaged region. The largest variations occurred in RBM absorbed doses of femora, pelvis, and sacrum. These variations were mainly due to the differences in imaging region length and the position of the isocentre. Hospital M4 wide and narrow protocols for prostate imaging highlight the effect of imaging region length to RBM absorbed doses. The protocols differed only by their imaging region lengths (27.7 cm vs 13.5 cm). Hospital C used 41 cm imaging region length which was the widest of all simulated protocols, explaining notably high doses. The protocol also had an exposure time of 26.4 s when the typical exposure time was 18 s. Hospital C caused similar absorbed doses to hospitals D1 and H1 with comparable current-time-product values for organs located near the beam centre (e.g., 34 mGy vs. 27 mGy vs. 26 mGy for the prostate, Table 5). Slightly larger doses for hospital C than D1 or H1 can be explained by increased scatter caused by a wider X-ray beam. Hospitals M4 and J had lowest tube current-time products (442 mAs and 252 mAs) and, accordingly, the mean organ doses are the lowest of all simulated protocols.

Mean absorbed doses to the stomach were well below 1 mGy per imaged fraction, except for hospital C, where the lengthy imaged region directly exposes the stomach unlike other protocols. The calculated doses are in line with previous studies which also indicate significant organ absorbed doses from different imaging protocols.^9,24,25^

The MC simulations were validated with measurements in an anthropomorphic phantom. Generally, simulations and measurements agreed within uncertainties in areas that were homogeneous soft tissue material. In three measurement positions the difference cannot be explained by uncertainties only. In these cases, the adjacent bony structures and differences between the detector positions and ROIs in the simulation caused deviations - the regions where simulated doses were determined probably overlapped more bony structures than MOSFET measurements, therefore increasing the simulated dose relative to measurements.

Across the 14 European countries (35 hospitals) included in the survey for this study, significant variations in the approach to the patient dose from imaging were found, and there is room to enhance the optimization of these doses during radiotherapy treatments. Among the institutions, only three reported using national protocols for the patient dose assessment.

The significant reduction in patient organ doses can easily be achieved e.g. if imaging frequency is reduced, and the X-ray tube current-time product is reduced from the factory preset value. This cumulative organ dose reduction can reach hundreds of mGy. However, the lack of recommended protocols and guidance, as noticed by ESTRO, who in their recent publication provide practical advice for optimising and maintaining CBCT imaging protocols for the needs of local procedures and patient populations, may slow down this development.^5^ In this study, less than half of the hospitals have optimized their protocols either by reducing the FOV, number of projections, or X-ray tube current. All hospitals use the bowtie filters to reduce dose to organs located off the X-ray beam centreline. The importance of regular and appropriate quality assurance of CBCT devices is highlighted by their frequent use at hospitals.

The results of the survey reflect high workload of radiotherapy departments. There is an overall agreement among clinicians on the importance of quantitative consideration for the imaging dose when updating patient setup protocols. As an example, a clinical decision to reposition the patient and retake images often represents a balance between an ideal setup, time that the patient spends on the treatment table and the imaging dose. Analysing the latter will improve the decisionmaking process.

With new developments in radiotherapy becoming more widely available, there is an increasing demand to optimize radiation doses used for treatment more efficiently. As a result, the increase in high-quality, low-dose imaging will be obvious. Only 11 (31%) hospitals confirmed regular measurements and record-keeping doses for their imaging modalities.

The majority of the reported treatment protocols involved imaging at every treatment fraction. Some clinics switch to weekly imaging after the first few fractions. However, it was noted that some clinics return to daily imaging in case of inconsistencies to avoid misalignment in dose delivery. For some of the localizations, the impact of absorbed dose from imaging may be even more significant than for those sites simulated in this work. For instance, treatments and imaging in the thoracic region does not only include radiation-sensitive female breasts, but evidence of radiation-induced cardiovascular diseases is growing.^23^ Overall, the dose to an individual organ depends on the combination of imaging parameters but key factors can be distinguished from our simulations: Besides the tube current–time product, the imaging region length is a principal factor as it directly influences what organs gain exposure. Increasing the imaging region length will increase mean absorbed doses to organs at the edges of the beam thus exposing more healthy tissue to ionizing radiation. This increase in organ dose is particularly focused on the femora, but other organs such as bladder can be affected as well. However, the image must show the necessary details to enable patient positioning and for that purpose the imaging region must be selected appropriately even if it produces additional dose to the patient. The frequency of positioning imaging is a major factor that affects the patient dose. If required positioning accuracy can be obtained with less than daily imaging, the dose saving can be substantial.

The increase of absorbed dose in bones also affects the dose to RBM which is particularly sensitive to ionizing radiation. The absorbed dose to RBM is further enhanced in kV-CBCT imaging due to photoelectric events in the surrounding bones at less than 200 keV photon energies.^26^ Dose to RBM could not be calculated directly in ICRP CRP since it has been incorporated into the spongious volumes of bones.^18,27^ Thus, in the present study the dose in RBM was estimated using the massabsorption coefficients ratios in the RBM and the surrounding bone.

The purpose of this study was to examine a variety of organ doses from kV-CBCT imaging protocols in use. Given the nature of the study, the 13% uncertainty in the simulated organ doses (16% for RBM) should be sufficient to make comparisons between different imaging protocols and resulting organ doses in ICRP CRP. However, the uncertainties in real patient anatomies could be larger than the values stated in this study.

The CTDI_w_ values were used to establish a tentative DRL values for patient positioning imaging in the pelvic area. However, in some of the countries that participated in the survey, limited CTDI_w_ data were available. Therefore, a more comprehensive and extensive data collection is needed to establish robust national and/or regional DRLs. Such an initiative would support the recommendations of the ICRP14 and further support the optimisation of patient doses in positioning imaging. The results of this study suggest that representative CTDIw values could be 17.7 mGy for pelvic imaging and 13.5 mGy for prostate imaging. However, careful analysis of the prerequisites for imaging is essential when setting DRLs, as different positioning techniques require varying imaging strategies, volumes, and image quality levels.

Patient positioning imaging is an essential part of IGRT, and it cannot be avoided. However, the frequency and dose of the imaging can be optimized to ensure acquisition of the necessary positioning information while minimizing patient organ doses. The results of this study indicate that formal imaging guidelines and protocols for IGRT with CBCT are largely lacking, with each clinic following its own practices. Consequently, there is substantial variation in mean organ doses among clinics, for example, prostate imaging protocols showed a range from 4 mGy to 26 mGy per fraction. Overall, there is considerable room for dose optimization. In some cases, the imaged region may be unnecessarily long, or the tube current-time product may remain at default factory settings without adjustment. Even though certain protocols have been optimized by vendors, manufacturers, or end users, cumulative mean organ absorbed doses from imaging can still reach several hundred mGy in the imaged region if imaging is performed at every treatment fraction (with mean organ doses per fraction potentially reaching 40 mGy). Most clinics reported conducting regular quality assurance of imaging equipment, including verification of dosimetry and positioning accuracy. In the future, a similar investigation could be extended to include the head and neck region, as well incorporate a female anatomic model. Intrafractional planar kV imaging was not covered in this study, and it should be considered in future studies to address the full radiation dose of the patient.

## References

[1] Thwaites D. (2013). Accuracy required and achievable in radiotherapy dosimetry: have modern technology and techniques changed our views?. J Phys Conf Ser.

[2] McNair HA, Franks KN, van Herk N. (2022). On Target 2: updated guidance for image-guided radiotherapy. Clin Oncol.

[3] Martin CJ, Kron T, Vassileva J, Wood TJ, Joyce C, Ung NM (2021). An international survey of imaging practices in radiotherapy. Phys Med.

[4] Djukelic M, Martin CJ, Abuhaimed A, Kron T, Gros S, Wood T (2025). Cone beam CT (CBCT) in radiotherapy: assessment of doses using a pragmatic setup in an international setting. Phys Med.

[5] Wilson LJ, Hadjipanteli A, Østergaard DE, Bogaert E, Brown KF, DeJong R (2025). Cone beam CT dose optimisation: a review and expert consensus by the 2022 ESTRO Physics Workshop IGRT working group. Radiother Oncol.

[6] Özseven A, Dirican B. (2021). Evaluation of patient organ doses from kilovoltage cone-beam CT imaging in radiation therapy. Rep Pract Oncol Radiother.

[7] Shah A, Aird E, Shekhdar J. (2012). Contribution to normal tissue dose from concomitant radiation for two common kV-CBCT systems and one MVCT system used in radiotherapy. Radiother Oncol.

[8] Siiskonen T, Alenius S, Seppälä T, Tikkanen J, Nadhum M, Ojala J. (2024). Cone beam CT doses in radiotherapy patient positioning in Finland - prostate treatments. Radiat Prot Dosimetry.

[9] Marchant TE, Joshi KD. (2016). Comprehensive Monte Carlo study of patient doses from cone-beam CT imaging in radiotherapy. J Radiol Prot.

[10] Olch AJ, Alaei P. (2021). How low can you go? A CBCT dose reduction study. J Appl Clin Med Phys.

[11] Ibbott GS. (2020). Patient doses from image-guided radiation therapy. Phys Med.

[12] Martin CJ, Gros S, Kron T, Wood TJ, Vassileva J, Small W (2023). Factors affecting implementation of radiological protection aspects of imaging in radiotherapy. Appl Sci.

[13] AAPM (2018). Image guidance doses delivered during radiotherapy: quantification, management, and reduction. Report of the AAPM Therapy Physics Committee Task Group 180. Med Phys.

[14] ICRP (2017). ICRP Publication 135: Diagnostic reference levels in medical imaging. Ann ICRP.

[15] Buckley JG, Wilkinson D, Malaroda A, Metcalfe P. (2018). Investigation of the radiation dose from cone-beam CT for image-guided radiotherapy: a comparison of methodologies. J Appl Clin Med Phys.

[16] CT imaging GmbH (2016). ImpactMC User Guide Version 1.20.

[17] Poludniowski G, Omar A, Bujila R, Andreo P. (2021). Technical Note: SpekPy v2.0 – a software toolkit for modeling x-ray tube spectra. Med Phys.

[18] ICRP (2009). ICRP Publication 110: Adult reference computational phantoms. Ann ICRP.

[19] ICRP (2007). ICRP Publication 103: The 2007 Recommendations of the International Commission on Radiological Protection. Ann ICRP.

[20] Johnson PB, Bahadori AA, Eckerman KF, Lee C, Bolch WE. (2011). Response functions for computing absorbed dose to skeletal tissues from photon irradiation – an update. Phys Med Biol.

[21] Lee C, Lee C, Shah AP, Bolch WE. (2006). An assessment of bone marrow and bone endosteum dosimetry methods for photon sources. Phys Med Biol.

[22] Brady SL, Kaufman RA. (2012). Establishing a standard calibration methodology for MOSFET detectors in computed tomography dosimetry. Med Phys.

[23] Belzile-Dugas E, Eisenberg MJ. (2021). Radiation-induced cardiovascular disease: review of an underrecognized pathology. J Am Heart Assoc.

[24] Ding GX, Duggan DM, Coffey CW. (2008). Accurate patient dosimetry of kilovoltage cone-beam CT in radiation therapy. Med Phys.

[25] Altergot A, Schürmann M, Jungert T, Auerbach H, Nüsken F, Palm J (2023). Imaging doses for different CBCT protocols on the Halcyon 3.0 linear accelerator – TLD measurements in an anthropomorphic phantom. Z Med Phys.

[26] Zankl M, Eakins J, Gómez Ros J-M, Huet C. (2021). The ICRP recommended methods of red bone marrow dosimetry. Radiat Meas.

[27] Zankl M, Eckerman KF, Bolch WE. (2007). Voxel-based models representing the male and female ICRP reference adult – the skeleton. Radiat Prot Dosimetry.

